# Disruption of proteome by an oncogenic fusion kinase alters metabolism in fibrolamellar hepatocellular carcinoma

**DOI:** 10.1126/sciadv.adg7038

**Published:** 2023-06-21

**Authors:** Solomon N. Levin, Michael D. Tomasini, James Knox, Mahsa Shirani, Bassem Shebl, David Requena, Jackson Clark, Søren Heissel, Hanan Alwaseem, Rodrigo Surjan, Ron Lahasky, Henrik Molina, Michael S. Torbenson, Barbara Lyons, Rachael D. Migler, Philip Coffino, Sanford M. Simon

**Affiliations:** ^1^ Laboratory of Cellular Biophysics, The Rockefeller University, 1230 York Avenue, New York, NY 10065, USA.; ^2^ Proteomics Resource Center, The Rockefeller University, 1230 York Avenue, New York, NY 10065, USA.; ^3^ General Surgery Division, Surgery Department, Hospital Nove de Julho, São Paulo, Brazil.; ^4^ Lahasky Medical Clinic, Abbeville, LA 70510, USA.; ^5^ The Fibrolamellar Registry, New York, NY 10028, USA.; ^6^ Division of Anatomic Pathology, Mayo Clinic, Rochester, MN 55904, USA.; ^7^ Department of Chemistry and Biochemistry, New Mexico State University, Las Cruces, NM 88003, USA.

## Abstract

Fibrolamellar hepatocellular carcinoma (FLC) is a usually lethal primary liver cancer driven by a somatic dysregulation of protein kinase A. We show that the proteome of FLC tumors is distinct from that of adjacent nontransformed tissue. These changes can account for some of the cell biological and pathological alterations in FLC cells, including their drug sensitivity and glycolysis. Hyperammonemic encephalopathy is a recurrent problem in these patients, and established treatments based on the assumption of liver failure are unsuccessful. We show that many of the enzymes that produce ammonia are increased and those that consume ammonia are decreased. We also demonstrate that the metabolites of these enzymes change as expected. Thus, hyperammonemic encephalopathy in FLC may require alternative therapeutics.

## Teaser

An oncogenic fusion kinase alters key pathways of mitochondrial metabolism in fibrolamellar hepatocellulalar carcinoma.

## INTRODUCTION

Fibrolamellar hepatocellular carcinoma (FLC) is a primary liver cancer that predominantly affects adolescents and young adults (*1*). The 5-year survival rate has been reported to be 33.6 to 42.6% (*2*, *3*), although a recent study with many patients with a molecular confirmation of diagnosis found a higher 5-year survival of 62.2% (*4*). There is no approved systemic therapy for FLC. FLC is classified as a subtype of noncirrhotic hepatocellular carcinoma (HCC) (*5*). As a result, patients with FLC are treated with therapies for HCC, although FLC has a distinct molecular pathology. One severe complication of FLC is hyperammonemic encephalopathy that is associated with high mortality (*6*, *7*).

FLC is driven by dysregulation of protein kinase A (PKA) (*1*). In most patients, a somatic deletion of ~400 kb on one copy of chromosome 19 fuses the first exon of a heat shock protein cofactor (*DNAJB1*), with the second through final exons of the catalytic subunit of PKA (*PRKACA*) (*8*). Expression of a kinase active DNAJB1::PRKACA fusion protein results in formation of liver tumors (*9*, *10*) that exhibit many of the histological features of FLC (*11*), including pale bodies, eosinophilic cytoplasm, and steatosis, and recapitulate many of the changes of the transcriptome observed in patients’ tumors (*9*, *12*). FLC is oncogenically addicted to the oncokinase: Elimination of the *DNAJB1::PRKACA* transcript with short hairpin RNA results in cell death (*13*).

Although FLC is a relatively rare cancer (1 in 5 million), it has a well-defined histopathology and a consistent molecular etiology (*1*). As result, the changes in the coding and noncoding transcriptome from different patients cluster together and away from the transcriptome of the adjacent normal, and it also clusters away from the transcriptome of other liver tumors (*12*, *14*). The uniformity of FLC makes it a favorable tool for exploring the pathogenicity of cancer. Several critical advances in cancer biology have come from the study of such rare but well-defined cancers. The precision of the characterization promotes the elucidation of general principles. While the significance of tumor suppressor genes is now widely appreciated for many cancers, they were found from the study of an equally rare cancer, retinoblastoma (*15*, *16*). Mutations in isocitrate dehydrogenases are now explored for many cancers, but they were first characterized in a rare glioblastoma (*17*).

Here, we used mass spectrometry (MS) to interrogate the proteome and metabolome of FLC to search for changes that explain some of the pathology. The results expand our catalog of consistently altered proteins in FLC, many of which are in enzymes that affect the metabolism of drugs. These changes help elucidate the sensitivity of FLC in high-throughput drug-repurposing screens (*18*, *19*). Other altered enzymes are in the mitochondria and are involved in glycolysis and metabolism of ammonia. The consistent alterations of glycolytic enzymes may allow for easier elucidation of metabolic dependencies in cancers. In addition, changes in metabolites are consistent with the observed alterations of enzymes involved in the metabolism of ammonia. The often-observed hyperammonemic encephalopathy in FLC is likely the confluence of both production of ammonia by FLC and a deficiency in two major pathways of consumption. The results suggest alternative approaches to the treatment of hyperammonemia in FLC.

## RESULTS

FLC tumor and adjacent nontransformed (normal) liver samples from 11 patients were quick-frozen upon resection in the operating room to minimize postsurgical changes. Although we analyzed tissue frozen rapidly after resection, there is potential for changes in the transcriptome and proteome during anesthesia, vessel ligation, and resection. Therefore, these studies were limited to contrasting the FLC tumor to the adjacent normal of the same patient sample, as both may be subject to similar artifacts. Two independent MS-based approaches were used to characterize the proteins: label-free quantification (LFQ) and tandem mass tag multiplexing (TMT). Each of the techniques has its strengths and limitations, and we probed for conclusions consistent between the two. TMT is considered to be more sensitive than LFQ, but it does not have as wide a dynamic range as LFQ, whose quantification is a fairer representation of protein levels in the sample (*20*). TMT allowed us to analyze 16 samples in a single experiment: paired tumor and adjacent non-neoplastic liver samples from eight patients. Following digestion, reduction, and alkylation of samples, peptides were TMT-tagged and combined into a single sample for analysis. For the LFQ analysis, we also studied eight paired samples, but from three additional patients, allowing us to test multiple samples from different tissue regions of the same patient. Subsequent steps of sample processing and data analysis are detailed in Materials and Methods. All MS runs and analysis were run blind to sample identity. For individual proteins detected by both LFQ and TMT from different patients, the *r*^2^ = 0.8 (fig. S1).

**Fig. 1.**
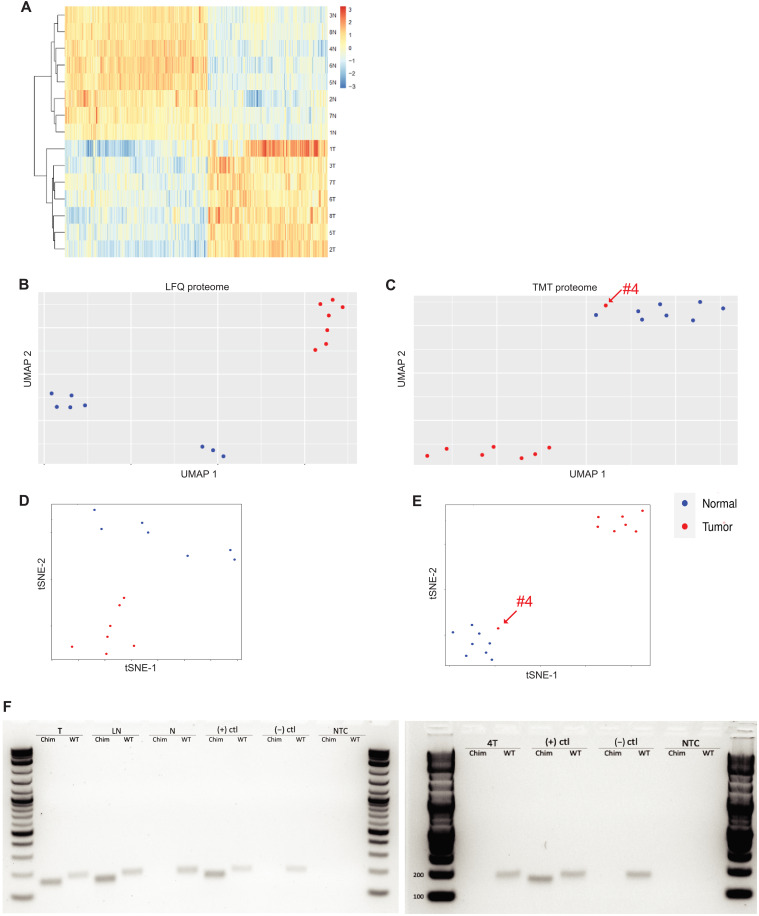
Proteomics of FLC. Hierarchical clustering of proteins in FLC and normal samples; each row represents the sample labeled to the right of the row. The top cluster are the normal samples; the bottom are the tumor samples. (**A**) Unsupervised hierarchical clustering of all proteins that are statistically up-regulated or down-regulated in the TMT detection. (**B** and **C**) UMAP analysis indicates two clusters of the proteome by LFQ (B) and TMT (C) and tSNE clustering by LFQ (**D**) and TMT (**E**). Sample 4T in the TMT plots is marked at the arrow in the proteome by TMT. (**F**) RT-PCR to test whether a tissue sample had the *DNAJB1::PRKACA* fusion. Chim is RT-PCR with primers probing for chimeric *DNAJB1::PRKACA* (expected amplicon: 160 bp), and WT is RT-PCR with primers probing for wild-type *PRKACA* (expected amplicon: 184 bp). From left to right: T is the tumor sample, LN is a metastatic lymph node, N is the adjacent normal tissue, (+)ctl is RNA from a tumor of patient with FLC, (−)ctl is RNA from a non-neoplastic liver sample from a non-FLC patient, NTC is a no RNA template control, and 4T is the tumor sample from patient #4.

An unsupervised hierarchical clustering of all of the proteins that were significantly up- or down-regulated consistently in TMT or LFQ found that the tumor samples clustered together and away from all of the normal samples (Fig. 1A). When LFQ was used to assess the proteome, all eight tumor samples clustered together and away from the proteome from the adjacent normal. This clustering was observed whether the assessment was by uniform manifold approximation and projection (UMAP; Fig. 1B) or *t*-distributed stochastic neighbor embedding (tSNE) (Fig. 1D). The clustering was observed for different samples taken from the same patient as well as samples from different patients. When TMT was used to assess the proteome from eight other patients, the tumor tissues for seven patients clustered together, and apart from the proteome of the adjacent non-neoplastic liver. This clustering was observed whether analyzed by UMAP (Fig. 1C) (*21*, *22*) or by tSNE (Fig. 1E) (*23*, *24*). An exception was the tumor of patient #4, which clustered with non-neoplastic liver samples.

Two different approaches were taken to validate the original diagnosis of FLC. For assessment of the chimeric *DNAJB1::PRKACA* transcript in patient tissue samples, RNA was extracted and reverse transcription polymerase chain reaction (RT-PCR) performed using two different pairs of primers: A reverse primer from the second exon of *PRKACA* and a forward primer from either the first exon of *DNAJB1*, which would indicate a fusion transcript, or the first exon of *PRKACA*, which would indicate the native transcript (Fig. 1F). Primary or metastatic FLC tumor tissue yields PCR products for both the *DNAJB1::PRKACA *chimera transcript [160–base pair (bp) amplicon] and native *PRKACA* (184-bp amplicon). In contrast, the adjacent non-neoplastic liver (normal) or negative controls (a non-FLC, HCC tumor) yield only the PCR product of wild-type *PRKACA*. We did this validation test on all tissue samples and found that the *DNAJB1::PRKACA* 160-bp amplicon was absent in the 4T tumor sample but present in all the tumors. A second assessment of the diagnosis of FLC was by histological analysis. Hematoxylin and eosin (H&E) stained sections of 270 samples in our collection were sent to a board-certified pathologist (M.S.T.) who was blinded to their identity. The tumor from patient #4 was judged to be a hepatic adenoma, most likely inflammatory subtype, and not FLC. This substantiates the validity of our proteome analysis in distinguishing a distinctive molecular landscape of FLC. For all future analyses, sample 4T was excluded. Patient #4 was one of our first samples, taken before we performed routine PCR validation, and was initially diagnosed elsewhere as FLC. The results demonstrate the importance of a molecular test for diagnosis.

**Fig. 2.**
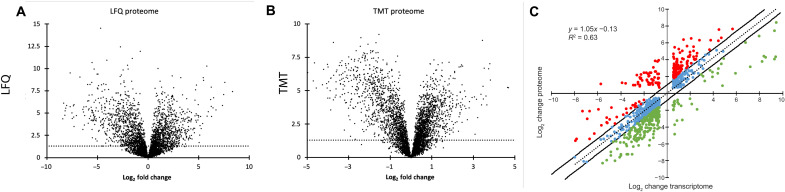
Analysis of the proteome. (**A** to **B**) Volcano plots of differential protein expression. Each point represents a protein assessed by (A) LFQ or (B) TMT. The *x* axis is the log_2_ fold change in the tumor relative to the adjacent normal. The *y* axis is the −log(FDR). Points above the dotted line are those that are significant at an FDR of ≤ 0.05. The proteins on the right have a higher expression in FLC tumor; those on the left have a higher expression in normal liver. (**C**) The differential expression of the transcriptome plotted as a function of the differential protein expression, as assessed by LFQ. Dots in red are those where the proteome is increased relative to the transcriptome, and dots in green are those where the proteins are decreased relative to the transcriptome (|log_2_| fold change ≥ 0.585).

In the LFQ analysis, 4098 different proteins were detected (table S1). Of these, 20.6% (846) were significantly increased, and 21.4% (879) were significantly decreased in tumor [|log_2_| fold change ≥ 0.585 (fold change ≥ 1.5) and false discovery rate (FDR) ≤ 0.05] relative to the adjacent normal (Fig. 2A). TMT-MS detected 4650 different proteins in 15 samples (table S2). Of these, 17% (768 proteins) were increased, and 19% (879 proteins) were decreased in the FLC tumor relative to the adjacent normal [|log_2_| fold change ≥ 0.585 (fold change ≥ 1.5) and FDR < 0.05] (Fig. 2B).

An increase in polypeptide detection for a protein in a tumor sample could reflect an increase in transcription, an increase in translation, or a decrease in proteolytic degradation. We previously performed a characterization of the FLC transcriptome on a separate patient set that showed little variability between patients (*12*). The levels of many proteins found to be altered in the FLC tumor, as assessed by MS, showed a linear correlation level (*r*^2^ = 0.63) with previously determined changes in transcriptome expression (Fig. 2C) (*12*). This suggests that for many of these proteins, the level of the protein is predominantly determined by transcript abundance and less so by posttranscriptional or posttranslational regulation. However, there are some proteins that deviate from this line. Some are distinctly increased (red) relative to the transcript level, which is consistent with either increased translation or decreased degradation. Some are decreased (green), consistent with decreased translation or increased proteolytic degradation.

Some of the deviations between transcriptome and proteome were in proteins involved in drug metabolism, such as the uridine 5′-diphosphate (UDP)–glucuronosyltransferase (UGT1A) family. These add sugars to various molecules (bilirubin, therapeutics) to facilitate their export via the adenosine 5′-triphosphate (ATP)–binding cassette membrane transport (ABCC2) for excretion into the bile. In the MS, UGT1A8, UGT1A9, and UGT1A10 are decreased at the protein level (log_2_ = −5.43). Deviations were also observed in metabolic enzymes. Hexokinase 2 (HK2) is increased at the transcriptome log_2_ = 2.34 but is increased at the protein level log_2_ = 4.97. Glutaminase (GLS) is increased at the transcriptome level log_2_ = 1.17 but increased at the protein level log_2_ = 4.73. These differences are of great potential significance for the cell. Thus, the proteomic data give a valuable and different perspective on the pathogenic alterations.

### Mitochondrial dysregulation

**Fig. 3.**
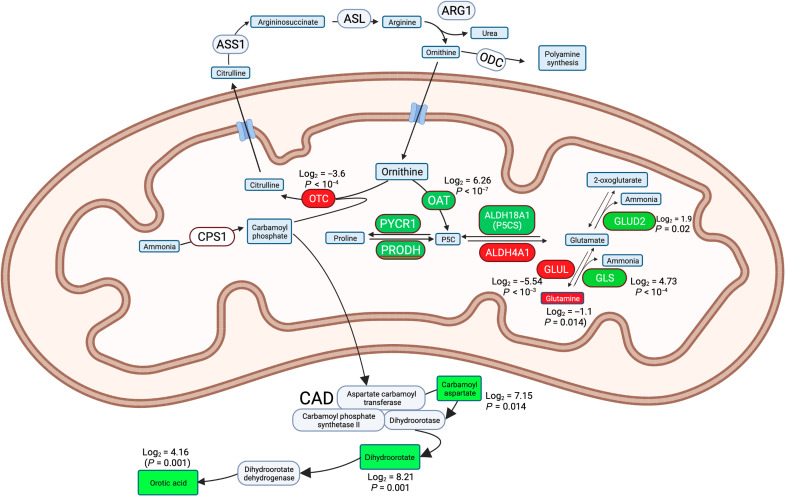
Pathways involved in metabolism of ammonia. Proteins are in ovals and metabolites are in rectangles. Those in green are proteins or metabolites increased in FLC cells, and those in red are decreased.

In our analysis, we noticed that many of the proteins showing the greatest changes between tumor and normal were mitochondrial proteins. To check this, we compared our dataset of proteins showing significant alterations with the MitoCarta3.0 database (*25*). This showed that a greater percentage of mitochondrial proteins were altered (36.6% LFQ and 33.3% TMT) compared to the total proteome (8.9% LFQ and 8.5% TMT). Many of the proteins altered are involved in the urea cycle and metabolism (Table 1 and Fig. 3). This includes enzymes involved in metabolism, such as HK2, malate dehydrogenase (MDH), pyruvate kinase M (PKM), triosephosphate isomerase (TPI1), and voltage-dependent anion-selective channel protein 2 (VDAC2). We probed for proteins in metabolism because mitochondrial metabolism is often disrupted in tumors (*26*–*28*) and mitochondrial metabolism is a frequent target for cancer therapy (*27*, *29*).

**Table 1.** Proteins involved in metabolism or ammonia metabolism. All proteome values are from LFQ. The transcriptome is from Simon *et al.* (*12*). NS, not significant.

Ammonia metabolism					
	Proteome		Transcriptome		
Gene name	Log_2_ change	FDR	Log_2_ change	FDR	Name
ALDH18A1	2.66	1.00 × 10^−2^	2.11	8.81 × 10^−16^	Delta-1-pyrroline-5-carboxylate synthetase (P5C)
CPS1	0.45	2.06 × 10^−1^	1.22	2.01 × 10^−8^	Carbamoyl phosphate synthetase 1
GLUD2	1.89	2.09 × 10^−2^	1.32	2.87 × 10^−5^	Glutamate dehydrogenase 2
GLS	4.73	6.57 × 10^−5^	1.17	1.17 × 10^−11^	Glutaminase
GLUL	−5.54	6.67 × 10^−4^	−2.33	1.72 × 10^−18^	Glutamine synthetase
OAT	6.26	1.23 × 10^−8^	3.56	3.94 × 10^−29^	Ornithine aminotransferase, mitochondrial
OTC	−3.63	1.24 × 10^−5^	−3.20	2.50 × 10^−4^	Ornithine carbamoyltransferase, mitochondrial
PYCR1	4.3	3.06 × 10^−3^	2.02	2.51 × 10^−6^	Pyrroline-5-carboxylate reductase 1, mitochondrial
SLC38A1	NS	NS	2.39	1.04 × 10^−32^	Glutamate sodium–coupled neutral amino acid transporter 10
**Metabolism**					
	**Proteome**		**Transcriptome**		
Gene name	Log_2_ change	FDR	Log2 change	FDR	Name
HK2	4.97	1.11 × 10^−6^	2.34	1.44 × 10^−7^	Hexokinase 2
MDH1	−0.67	6.53 × 10^−3^	NS	NS	Malate dehydrogenase I
MDH2	0.96	7.87 × 10^−4^	NS	NS	Malate dehydrogenase II
NDRG2	−2.70	5.22 × 10^−9^	−2.49	5.51 × 10^−18^	NMYC downstream-regulated gene 2
PKM	0.95	1.10 × 10^−2^	0.85	1.02 × 10^−2^	Pyruvate kinase M
TPI1	−0.44	9.25 × 10^−4^	0.06	NS	Triosephosphate isomerase
VDAC2	1.34	6.30 × 10^−8^	0.70	9.42 × 10^−3^	Voltage-dependent anion-selective channel protein 2

We focused on proteins in ammonia metabolism because hyperammonemic encephalopathy is commonly observed in FLC and is sometimes a cause of death in these patients (*6*, *7*, *30*, *31*). In liver disease, ammonia toxicity can result from liver failure or specific mitochondrial dysfunction (*32*). In mice, 70% of ammonia is cleared by the liver, with half (35%) cleared by urea cycle enzymes and half by glutamine synthetase (GLUL) (*33*). We had previously observed a number of proteins involved in ammonia metabolism to be significantly altered in the FLC transcriptome (*12*). Some of these are involved in consuming or producing ammonia. We specifically probed to see whether these proteins and their metabolites are altered in patient FLC tissue relative to the adjacent nontransformed tissue (Fig. 3 and Table 1).

One pathway consumes ammonia by conjugating it to carbonate via carbamoyl phosphate synthase 1 (CPS1), to form carbamoyl phosphate. Carbamoyl phosphate is normally rapidly combined with ornithine by ornithine carbamoyl transferase (OTC) into citrulline. OTC in the liver is a sink for 35% of systemic ammonia and feeds citrulline into the urea and citric acid cycle. Deficiencies in OTC are the most common urea cycle disorder in humans who present with hyperammonemia (*34*). In FLC, the ornithine pool is expected to be decreased because ornithine aminotransferase (OAT), whose transcription is stimulated by PKA (*35*), is increased in FLC (log_2_ = 6.26). Importantly, OTC is decreased in FLC (log_2_ = −3.63), further reducing the ability to consume carbamoyl phosphate. In OTC deficiency, the carbamoyl phosphate diffuses out of the mitochondria and is converted in the cytosol to carbamoyl aspartate (*36*, *37*), which is converted in turn to dihydroorotate and then to orotic acid. Elevated orotic acid is diagnostic of OTC deficiency (*34*, *38*).

The decreased levels of OTC in FLC and coincident observations of hyperammonemia mirror the build-up of ammonia seen in cases of genetic OTC deficiency. We probed this relationship further with an MS analysis of the metabolites in FLC and the adjacent normal tissue to test whether these changes in enzymes result in the predicted changes of reactants and products.

The metabolomics assessment found that the top three metabolites accumulated in FLC tissue were all metabolites known to accumulate in OTC deficiency. Carbamoyl aspartate increased in the FLC tumor (log_2_ = 7.15; Table 2). Similarly, there was an increase in the downstream product dihydroorotate (log_2_ = 8.21) and in the downstream metabolite orotic acid (log_2_ = 4.16) in FLC tissue. In contrast, non-FLC patients who have defects in the enzymes upstream, CPS1 or *N*-acetylglutamate synthase, do not have elevated levels of orotic acid. To test for increased orotic acid in the urine of patients, we partnered with The Fibrolamellar Registry (http://fibroregistry.org), a patient-run medical registry. Their records had 10 patients with reported diagnoses of hyperammonemic encephalopathy. Four among these patients had reported urine test results, and all four had elevated orotic acid reported in their medical records (range, 10.2 to 250 mmol/mol creatinine). In the literature, we found five more patients with FLC with hyperammonemic encephalopathy with values of orotic acid in the urine of 238 mmol/mol creatinine (*6*) to 673 mmol/mol creatinine (*39*) (normal range, 0.2 to 1.5 mmol/mol of creatinine).

**Table 2.** Metabolite profiling of FLC tumor samples relative to the adjacent nontransformed tissue. Heatmap indicates the changes in metabolite abundance (log_2_ fold change, scale: red to blue, −2 to 2). Statistics: two-tailed paired two-sample *t* test, *n* = 5.

Metabolite name	Fold change log_2_ transformed (*P* ≤ 0.05)	Paired *t* test (*P* value)	Fold change (tumor:normal)
Dihydroorotate	8.21	0.0011	296
Carbamoyl aspartate	7.15	0.014	142
Orotic acid	4.16	0.0011	17.9
Dihydrobiopterin (BH2)	2.69	0.010	6.4
Uracil	2.38	0.0038	5.2
5-Deoxy-5-(methylthio)adenosine	2.32	0.043	5.0
Hypotaurine	1.94	0.022	3.8
*N*-acetyl-d-glucosamine	1.85	0.034	3.6
Cystine	1.50	0.042	2.8
Pantothenate	1.40	0.016	2.6
Hypoxanthine	1.23	0.0068	2.3
γ-Aminobutyric acid	1.14	0.0017	2.2
Pseudouridine	1.02	0.023	2.0
Pyruvate	0.92	0.031	1.9
2-Hydroxybutyrate	0.84	0.038	1.8
Lysine	0.72	0.047	1.6
*N*^6^-acetyllysine	0.58	0.043	1.5
Thiamine	−0.48	0.050	0.7
Pipecolic acid	−0.49	0.0060	0.7
Nicotinamide adenine dinucleotide (oxidized form)	−0.94	0.022	0.5
Glutamine	−1.10	0.014	0.5
Reduced form of nicotinamide adenine dinucleotide	−1.16	0.025	0.4
*N*-acetylcysteine	−1.20	0.021	0.4
Cystathionine	−1.22	0.029	0.4
Adenosine 5′-diphosphate	−1.25	0.0064	0.4
Mannose-1-phosphate/mannose-6-phosphate	−1.28	0.036	0.4
Glycerol-3-phosphate	−1.44	0.0059	0.4
β-Alanine	−1.46	0.012	0.4
Coenzyme A	−1.52	0.023	0.3
Glucose-6-phosphate	−1.53	0.021	0.3
Phosphocreatine	−1.54	0.040	0.3
Carnitine	−1.58	0.013	0.3
Ribose-5-phosphate	−1.63	0.0023	0.3
Nicotinamide adenine dinucleotide phosphate	−1.74	0.015	0.3
*N*-acetylglutamine	−1.88	0.015	0.3
Sedoheptulose-7-phosphate	−2.11	0.0049	0.2
ATP	−2.12	0.0005	0.2
Propionylcarnitine	−2.13	0.0033	0.2
Inosine 5′-monophosphate	−2.20	0.021	0.2
Glucose	−2.22	0.032	0.2
Glyceraldehyde-3-phosphate	−3.61	0.028	0.1

The second major pathway in the liver that is responsible for clearance of 35% of systemic ammonia uses GLUL to convert ammonia plus glutamate into glutamine. GLUL has been shown to play a critical role in detoxification of ammonia (*33*). GLUL is decreased in FLC (log_2_ = −5.54) (Table 1). Genetic deletion of liver GLUL in mice is sufficient to produce hyperammonemia (*32*). These results demonstrate that there are defects in the two major pathways for detoxification of ammonia by the liver: the introduction of ammonia into the urea cycle by OTC and the consumption of ammonia by GLUL.

The pathway that reverses the action of GLUL generates ammonia from glutamine using GLS. This enzyme is increased in the FLC tissue (log_2_ = 4.73). This increase in GLS suggests that increased production of ammonia is expected in FLC at the expense of glutamine. The analysis of the metabolome shows glutamine to be decreased in FLC tissue relative to the adjacent nontransformed tissue (log_2_ = −1.1) (Table 2). Thus, compared to the normal liver, FLC tissue may both fail to consume ammonia and produce excess ammonia. Consistent with this possibility, we have observed three patients with FLC in whom ammonia levels decreased 50% in the first 10 days following resection [one of these is shown in fig. S2, and some were published (*6*, *40*, *41*)].

### Confirmation of MS proteomics by immunofluorescence

**Fig. 4.**
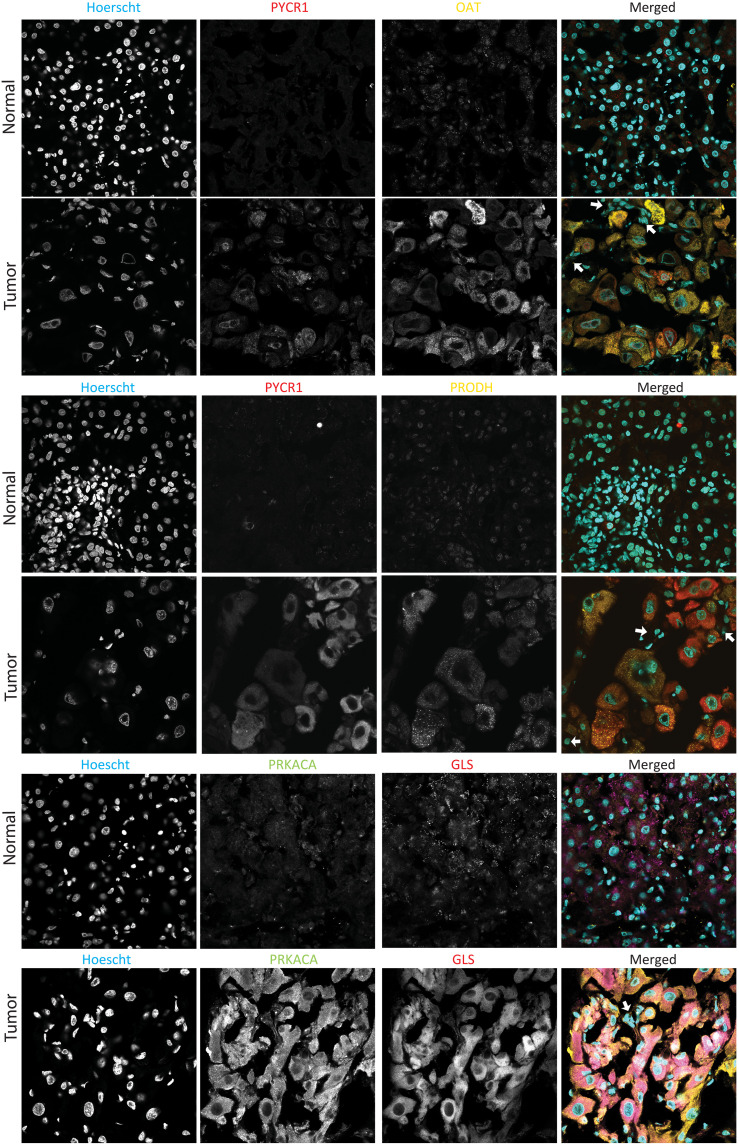
Immunofluorescence of mitochondrial enzymes. Probing of levels of PYCR1, PRODH, OAT, PRKACA, and GLS in FLC tumor and adjacent normal tissue. As is typical for FLC, the tumor cells are much larger than the normal hepatocytes. In the merged figure, the stromal cells, which are easily resolved by their smaller nuclei, are marked with an arrow. Each field is 212 μm on a side.

The observations on the proteome by MS report global changes in cell populations but do not provide information about tissue distribution. The alterations of the proteome may be in only one cell type (stromal, tumor, or normal), or the changes may only be in a restricted subset of cells. To examine this in patient tissue samples, we analyzed FLC tumor and adjacent normal tissue by immunofluorescence. Mitochondrial proteins increased in the tumor by MS proteomic analysis also increased in immunofluorescence in patient tumor cells relative to normal cells. Some examples are pyrroline-5-carboxylate reductase 1 (PYCR1), OAT, proline dehydrogenase 1 (PRODH), and GLS (Fig. 4 and fig. S3), all of which are involved in metabolism of ammonia. The increases in fluorescence for all four enzymes were relatively uniform in the FLC cells in multiple fields from the same slide and independent slides from the same patient and from three different patients. Notably, this increase in fluorescence is seen in the tumor tissue in the cells that are characteristically FLC tumor cells (large, polygonal) and expressing high levels of PRKACA and not in the stromal cells (Fig. 4, arrows). In addition, the increase is roughly similar in all FLC tumor cells, indicating that it is not the consequence of a few aberrant cells but true of all FLC cells.

## DISCUSSION

In this study, MS-based proteomics and metabolomics revealed distinct changes in the proteome in FLC tumor tissue compared to adjacent nontransformed liver tissue. These changes elucidate some critical mechanistic details about FLC. Two immediate issues are of consequence to patients. First, the results provide an explanation for why some previously used therapeutics showed such unexpected high efficacy against FLC in a high-throughput agnostic screen (*19*). Second, the results provide a mechanism for hyperammonemic encephalopathy, a frequent cause of death in FLC, and explains why conventional therapies have not worked, and why some alternatives are showing greater efficacy. Two additional insights contribute to understanding the pathogenesis of FLC. First, the results demonstrate how glycolysis is altered in FLC. Second, the results demonstrate that FLC is a very uniform disease. The changes in the proteome were consistent from patient to patient and segregated away from the adjacent normal tissue. The proteome also segregated away from that of other liver pathologies. This affirms the distinct nature of FLC.

### The proteome and drug efficacy

In previous agnostic drug–repurposing screens, there were some therapeutics hits that had not been predicted to be efficacious for FLC (*18*, *19*). These did not share a common target, and, thus, their efficacy was unexpected. One of the top hits was 7-ethyl-10-hydroxycamptothecin (SN38), the active metabolite of irinotecan (*18*, *19*). Liver cells modify SN38 by adding a sugar using the enzyme UDP glucuronosyltransferase 1 family (*42*). MS could not distinguish among UGT1A7, UGT1A8, UGT1A9, and UGT1A10, but, together, they are decreased at the protein level (log_2_ = −5.43). We have previously observed UGT1A1 to be decreased at the transcriptome level (log_2_ = −1.68) (*12*). This same enzyme is responsible for export of histone deacetylase (HDAC) inhibitors, which would further broaden the therapeutic window between FLC cells and normal liver for both irinotecan and HDAC inhibitors (*19*). There are numerous cancers that resist multiple therapeutic agents because of their increased ability to metabolize or export agent transporters (*43*, *44*). Here, we see the opposite and favorable result: FLC’s enhanced sensitivity as the result of an inability to metabolize agents, which therefore accumulate.

### Proteomics and energetics

A key to FLC pathogenesis may come from the finding here that FLC patient samples showed increases in many proteins involved in glycolysis (Table 1). An alteration in the number of mitochondria in FLC has been previously reported (*45*). Glycolysis is of interest in light of growing evidence that the pathogenesis of many cancers requires metabolic processing alterations. For example, PKM (increased in FLC tumor log_2_ = 0.95) is often a rate limiting enzyme in glycolysis, driving tumorigenesis. PKM has been proposed as both a diagnostic marker and as a therapeutic target (*46*). HK2 (increased in FLC log_2_ = 4.97) is required for tumor initiation and maintenance (*47*) and is critical for the homeostasis of glucose in cells. In mouse models, HK2 has been shown to be essential for initiation and continued maintenance of K-Ras–driven lung cancer and ErbB2-driven breast cancer (*47*). In adult humans, it is primarily expressed in the heart, adipose tissue, and muscle. However, a recent survey found that it overexpressed in 33 cancers in The Cancer Genome Atlas (*48*). Thus, there is growing interest in developing therapeutics that selectively target these pathways.

### Proteomics and hyperammonemic encephalopathy

Many of the alterations in the proteome of FLC tumor are in the mitochondrion, including enzymes involved in the interaction between ammonia metabolism and the urea cycle. A recurrent clinical problem in FLC is hyperammonemic encephalopathy (*6*, *30*, *40*, *41*, *49*–*53*). Alterations in the proteome of key enzymes in ammonia metabolism such as OTC, OAT, PYCR1, GLUL, GLS, glutamate dehydrogenase 2 (GLUD2), the glutamate solute carrier family 38 member 1 (SLC38A1), and aldehyde dehydrogenase 18 family member A1 (ALDH18A1) were seen in tumors of patients with FLC relative to the adjacent nontransformed tissue. The generation and consumption of ammonia is driven by pathways we found altered in FLC cells. The liver accounts for 70% of all systemic ammonia metabolism. Half of ammonia consumption by the liver is through CPS1, which catalyzes ammonia and bicarbonate into carbamoyl phosphate. Then, the enzyme OTC combines it with ornithine to make citrulline. In FLC, this pathway is affected at several points. First, in our original whole-genome sequencing, we found mutations in CPS1 in 2 of 10 patients (*45*). Second, OTC is decreased at the protein level (log_2_ = −3.63), reducing the ability to use ornithine to remove ammonia from the pool. We had previously reported a decrease in OTC (log_2_ = −3.2) at the transcriptome level (*12*). This would lead to an excess of carbamoyl phosphate and inhibit ammonia consumption. The activity of OTC is further decreased by the overexpression of the enzyme OAT (log_2_ = 6.26) that metabolizes free ornithine. This reduction of free ornithine would further reduce the activity of residual OTC. A second independent pathway consumes ammonia through incorporation into glutamate, by GLUL, to form glutamine. This pathway quantitatively accounts for the other half of liver-based ammonia metabolism. The enzyme GLUL is decreased at the protein level in FLC (log_2_ = −5.54). Unexpectedly, glutamine was reduced by 50% in the FLC cells. Given the large amount of glutamine in the body, we did not expect that the reduction of glutamine production in FLC cells would have a marked effect. A clue came from examining the proteome of the reverse pathway, whereby GLS generates ammonia from glutamine. GLS is significantly up-regulated (log_2_ = 4.73), which can explain the source of the glutamine loss in these cells. A third pathway that would lead to hyperammonemia is through GLUD2, increased in FLC (log_2_ = 1.9), which makes glutamate into ammonia and 2-oxoglutarate. How does the DNAJB1::PRKACA protein lead to these changes? It has been proposed that aurora kinase A expression increases *myc* expression that, in turn, suppresses *OTC* transcription (*7*). Alternatively, the increased PKA activity may directly suppress *OTC* transcription. In a genome-wide analysis of the adenosine 3′,5′-monophosphate response element–binding protein (CREB), PKA stimulation with forskolin, reduced transcription of *OTC* and a CREB response element resides in the *OTC* promoter (*54*).

The observations of both the proteome and metabolome suggest that there are three pathways that contribute to FLC hyperammonemic encephalopathy. FLC has been described as an “acquired” ornithine transcarbamylase deficiency (*6*, *7*, *31*, *39*, *50*). In other examples of OTC deficiency generated by mutations (*34*, *38*), excess carbamoyl phosphate diffuses out of the mitochondria and is then metabolized. We observed that each of the metabolic products is increased in FLC tumor: carbamoyl phosphate (increased log_2_ = 7.15), then dihydroorate (increased log_2_ = 8.21), and then orotic acid (increased log_2_ = 4.16). An examination of medical records of patients with FLC found all tested (n = 9) had levels of orotic acid increased 10-fold or more over the normal range. Unfortunately, most patients have not been tested for urine orotic acid levels, even with the onset of hyperammonemic encephalopathy. Consequently, it may be prudent to routinely test patients with FLC for urine orotic acid and, thus, potential ammonia imbalance. The decrease in glutamine is consistent with both the decreased expression of GLUL-consuming ammonia and increased GLS-generating ammonia. The observation that surgical debulking of FLC reduces ammonia levels is consistent with production of ammonia by FLC cells. Our results suggest that high ammonia in FLC is unlikely due only to the failure of the liver to consume ammonia but a specific generation of ammonia by FLC, potentially explaining the ineffectiveness of lactulose (directed at trapping ammonia in the gut) in treating FLC. Therefore we, and others, have turned to the use of scavengers for ammonia such as sodium benzoate and arginine (*6*, *7*, *31*, *39*, *40*, *50*, *51*, *55*). These agents can target tissue and serum ammonia, unlike lactulose that removes only ammonia in the gut. The present proteome data help explain the pathological changes that necessitate these treatments.

The observations on the altered expression of proteins in the mitochondria of FLC tumors offer both tantalizing potential targets for therapeutics and the potential of prognostics for distinguishing which patients are at risk for hyperammonemic encephalopathy. The observed consistent alterations of specific glycolytic enzymes in FLC may allow for easier elucidation of metabolic dependencies in cancers.

## MATERIALS AND METHODS

### RT-PCR validation of the chimera

Under supervision of our Institutional Review Board approval (Rockefeller IRB#SSI-0797, SSI-0-798), consent was obtained from patients scheduled for tumor resection. For each patient, the diagnosis of FLC was confirmed both by the demonstration of the DNAJB1-PRKACA fusion transcript by RT-PCR and DNAJB1-PRKACA fusion protein by Western blot (*8*). RNA patient tumors and nontumor liver were extracted using RNeasy Mini Kit (QIAGEN). Liver and tumor tissues were embedded in optimal cutting temperature, and frozen curls of 10 μm were lysed with RNAeasy buffer. RNA concentration was measured using NanoDrop 2000c (Thermo Fisher Scientific). All RNA samples were diluted to an equal concentration for the reverse transcription reaction. The LunaScript RT SuperMix Kit (New England Biolabs) was used to convert RNA into cDNA according to the manufacturer’s instructions. Platinum PCR SuperMix High Fidelity (Invitrogen) was used for PCR with the following conditions for each reaction: 22.5 μl of supermix, 1.5 μl of cDNA, and 0.5 μl of each primer at 10 μM (final primer concentration, 200 nM). Reactions were performed on a C1000 Thermal Cycler (Bio-Rad) as follows: 2 min at 94°C, followed by 30 cycles of 30 s at 94°C, 30 s at 55°C, and 20 s at 68°C. The PCR product was run on a 2% agarose gel with SYBR Safe (Invitrogen) powered with a PowerPac (Bio-Rad) set at 100 V for 60 min. Gel was imaged using Gel Doc EZ imager (Bio-Rad). Primer sequences are as follows: *DNAJB1::PRKACA*, GCCGAGGAGAAGTTCAAGGA (forward) and CTGTGTTCTGAGCGGGACTT (reverse), expected amplicon: 160 kb; PRKACA, GAGCAGGAGAGCGTGAAAGAA (forward) and TCATGGCATAGTGGTTCCCG (reverse), expected amplicon: 184 kb.

### MS of patient tissue

#### Patient tissue processing

Tissue preparation protocol was modified from Mertins *et al.* (*56*). Liver samples for proteome or transcriptome analysis were placed on dry ice immediately after resection and stored at −80°C. For tissue lysis, small pieces (2 to 5 mm in diameter) were cut from the specimen and placed in Eppendorf tubes. The tube was placed in a liquid nitrogen cooled mini mortar and pestle set that accommodates Eppendorf tubes (Bel-Art, catalog no. H37260-0100). The tissue was pulverized over liquid nitrogen. Ice-cold lysis buffer [8 M urea, 75 mM NaCl, 50 mM tris (pH 8.0), and 1 mM EDTA] supplemented with cOmplete EDTA-free protease inhibitor (Roche), and PhosSTOP phosphatase inhibitor (Roche) tablets were added to the pulverized tissue. The tube was then vortexed at maximum speed for 15 s, incubated on ice for 15 min, and then vortexed again at maximum speed for 15 s. The sample was then spun at 30,000 relative centrifugal field (RCF) for 30 min. To avoid the lipid layer, the Eppendorf tube was punctured near the bottom (above the pellet), and the supernatant flowed into another Eppendorf tube until the lipid layer reached the level of the hole. All spins were performed at 4°C. The lysate was assayed for protein concentration using the bicinchoninicacid (BCA) assay (Pierce).

#### Digestion

Reduction and alkylation were carried out using dithiothreitol and indole-3-acetic acid. Proteins were precipitated with the chloroform/water/methanol method (*57*) to remove remnant lipids. Pellets were digested with lysyl endopeptidase (Wako) overnight at room temperature. The samples were further digested for 6 hours at room temperature, using sequencing grade modified trypsin (Promega). Peptides were then tagged with tandem mass tags from the TMTpro 16-plex Label Reagent Set (Thermo Fisher Scientific) following the manufacturer’s instructions.

#### SPS-MS3 analysis for peptides

Peptides were fractionated using a Dionex 3000 Ultimate loading pump equipped with a 2.1*150 mm 3.5-μm Xbridge C18 column (Waters). Solvent A consisted of 10 mM ammonium hydroxide (Sigma-Aldrich) in water (pH 10), and solvent B consisted of 10 mM ammonium hydroxide and 90% acetonitrile in water (pH 10). Peptides were separated across a 60-min gradient, and 96 fractions were collected and concatenated for a total of 24 fractions. Fractions were analyzed using a Fusion Lumos mass spectrometer with SPS-MS3 acquisition. Data were analyzed using Proteome Discoverer v.2.3. Spectra were queried against the human proteome with a 1% FDR. Eighty percent of SPS matches were required for a hit to be included.

#### Database searching

Acquired RAW files for the peptides were analyzed in the MaxQuant framework (v. 1.6.0.13). Spectra were queried against the human proteome and searched with a 1% FDR on both peptide spectrum matched (PSM) and protein level. Carbamidomethylation of C and phosphorylation (S, T, or Y) were applied as variable modifications. A maximum of five modifications was allowed on each peptide.

#### Data analysis

Initial data analysis was performed within the Perseus framework. Intensities were log_2_-transformed and normalized by subtraction of the median in each experiment. Missing values were imputed by low-abundant random signals. The resulting intensities were imported and further analyzed in R v4.0.2 and Rstudio v1.3.1073 using base R statistics. Significant differences in intensities were determined by Student’s *t* tests, and *P* values were adjusted with the Benjamini-Hochberg algorithm. Graphics were created with the ggplot2 package.

### Metabolomics

Flash-frozen tissue from FLC tumor and adjacent nontransformed tissue from five patients were manually ground to a powder in a N_2_(l)-chilled porcelain mortar and pestle. The tissue samples were extracted in cold 80% methanol containing 1 μM internal standards (MSK-A2-1.2, Cambridge Isotope Laboratories Inc.) at a ratio of 0.025 ml/mg of tissue. The samples were vortexed vigorously for 10 min, followed by centrifugation at 16,000 RCF at 4°C for 30 min. The supernatant (0.5 ml) was transferred into a prechilled Eppendorf tube, snap-frozen, and concentrated to dryness using a speed vacuum. After resuspension of the dried polar samples in 60 μl of cold 50% acetonitrile, a 5-μl aliquot of the supernatant was diluted into a high-performance liquid chromatography vial containing 45 μl of 50% acetonitrile. The diluted samples were vortexed vigorously for 10 s, and 5 μl was injected onto the liquid chromatography–MS in a randomized sequence. Polar metabolites were separated on a ZIC-pHILIC 150 × 2.1 mm (5 μm particle size) column (EMD Millipore) connected to a Thermo Vanquish ultraperformance liquid chromatography system and a Q Exactive benchtop orbitrap mass spectrometer equipped with a heated electrospray ionization probe as reported previously (*58*).

Relative quantitation of polar metabolites was performed using Skyline Daily (v 21.1.9.353) (*59*) with the maximum mass and retention time tolerance set to 2 parts per million and 12 s, respectively, referencing an in-house library of chemical standards. Missing values (<5%) were imputed using one-fifth of the minimum positive values detected for each metabolite. Metabolite levels (peak areas) were normalized to the median metabolite signal within each sample. Principal components analysis was performed, using MetaboAnalyst v 5.0 (*60*), on log_2_-transformed data (normalized to median metabolite signal). Metabolites that contained signals in four-fifth replicates (in at least one biological group) were compared using a paired *t* test.

## Notes

View/request a protocol for this paper from *Bio-protocol*.
