# Supplementary material for: Disruption of proteome by an oncogenic fusion kinase alters metabolism in fibrolamellar hepatocellular carcinoma
Source: Sci Adv. 2023 Jun 21;9(25):eadg7038. doi: 10.1126/sciadv.adg7038 (PMC10284549; doi:10.1126/sciadv.adg7038)
Supplement: Supplementary file 1 — Legends for tables S1 and S2 Figs. S1 to S3 [file sciadv.adg7038_sm.pdf]

Science Advances
AAAS

Supplementary Materials for

Disruption of proteome by an oncogenic fusion kinase alters metabolism in fibrolamellar hepatocellular carcinoma

Solomon N. Levin *et al.*

Corresponding author: Sanford M. Simon, simon@rockefeller.edu

Sci. Adv. **9**, eadg7038 (2023)
DOI: 10.1126/sciadv.adg7038

The PDF file includes:

Legends for tables S1 and S2
Figs. S1 to S3

Other Supplementary Material for this manuscript includes the following:

Tables S1 and S2

Supplement:

Supplemental Table 1:

Supplemental Table 1 – Proteins detected by TMT of patient samples - (see excel spreadsheet)

Supplemental Table 2 – Proteins detected by LFQ of patient samples- (see excel spreadsheet)

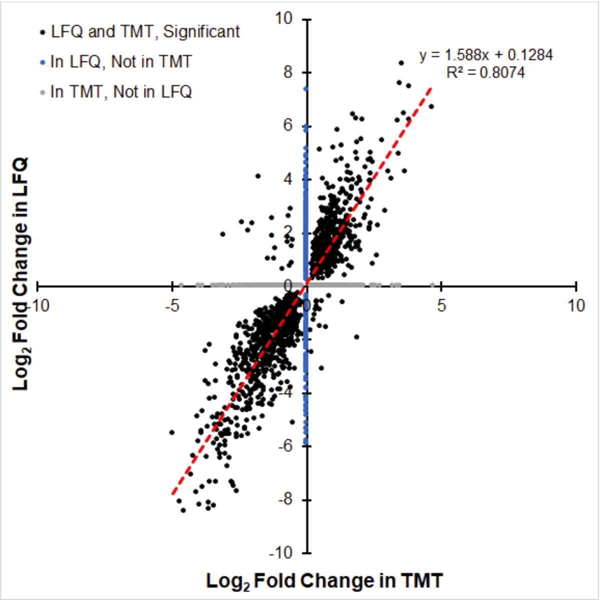

Supplemental figure 1: The quantification of LFQ as a function of the quantification of TMT for all of the proteins whose detection was significant with both techniques (black dots). The linear correlation coefficient is r^2=0.8. There are some peptides that are not detected well in LFQ or not detected well in TMT, so we have also plotted only those proteins detected by TMT (gray, along the x-axis) or only those proteins detected by LFQ (blue, along the y-axis). The TMT is more sensitive at the lower concentrations, but less sensitive at the higher levels, so the slope is > 1.

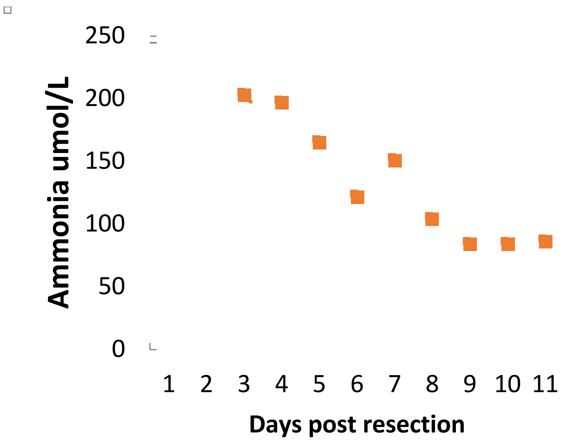

Supplemental Figure 2. Measurement of ammonia level in the blood of a patient in the days after surgical debulking.

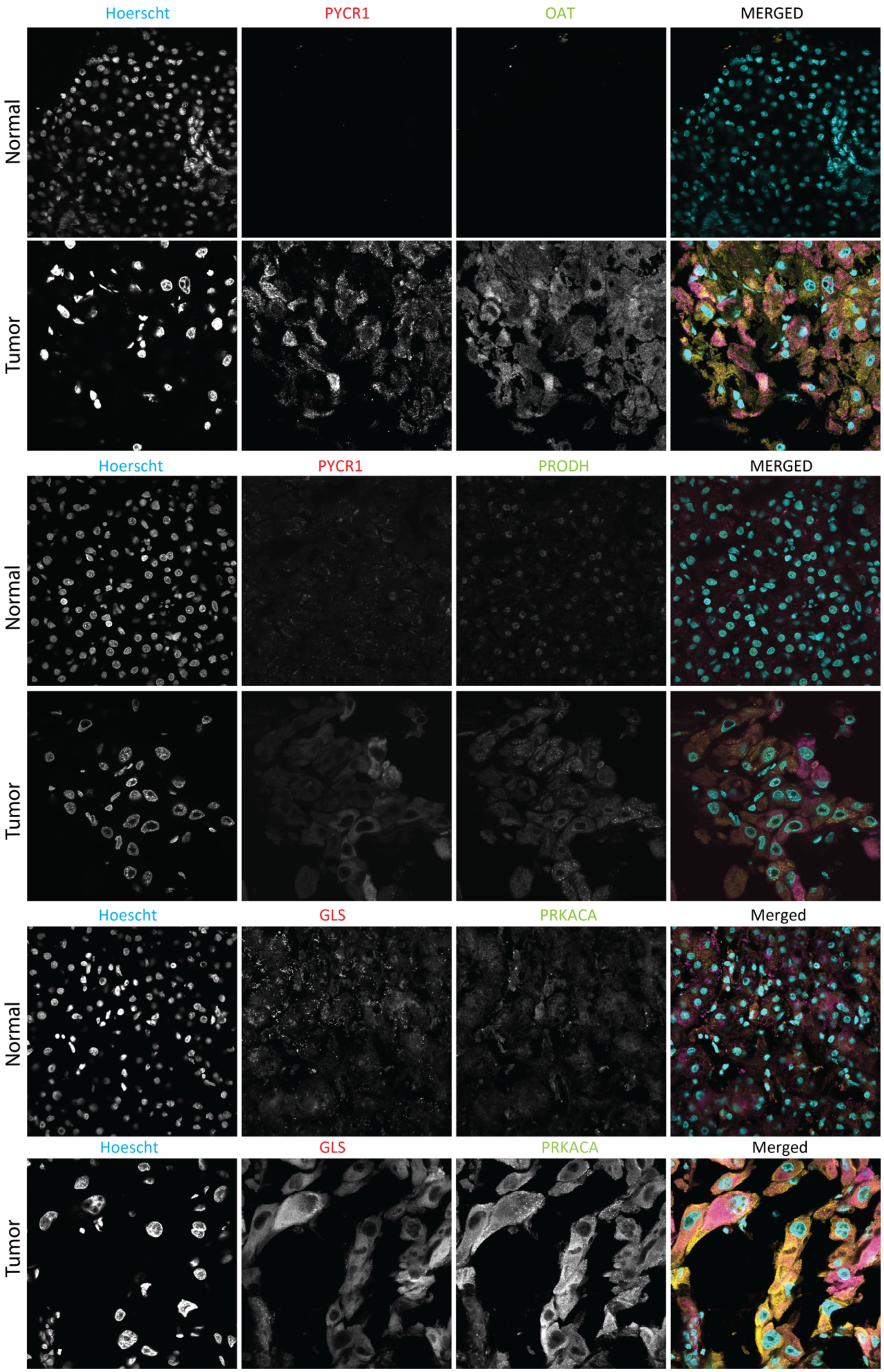

Supplemental figure 3. Immunofluorescence of mitochondrial enzymes (different fields/cells from Figure 4). Probing of levels of PYCR1, PRODH, OAT, PRKACA and GLS in FLC tumor and adjacent normal tissue. As is typical for FLC, the tumor cells are much larger than the normal hepatocytes. Each field is 212 μm on a side.
